# Challenges and opportunities for infection prevention and control in hospitals in conflict-affected settings: a qualitative study

**DOI:** 10.1186/s13031-021-00428-8

**Published:** 2021-12-20

**Authors:** Hattie Lowe, Susannah Woodd, Isabelle L. Lange, Sanja Janjanin, Julie Barnet, Wendy Graham

**Affiliations:** 1 Department of Infectious Disease Epidemiology, Faculty of Epidemiology and Population Health, The London School of Hygiene and Tropical Medicine, London, UK; 2 Present Address: Institute for Global Health, Univeristy College London, London, UK; 3 Health Unit, International Committee of the Red Cross, Geneva, Switzerland

**Keywords:** Infection prevention and control, Conflict, Healthcare associated infection, Health facility

## Abstract

**Background:**

Healthcare associated infections (HAIs) are the most frequent adverse outcome in healthcare delivery worldwide. In conflict-affected settings HAIs, in particular surgical site infections, are prevalent. Effective infection prevention and control (IPC) is crucial to ending avoidable HAIs and an integral part of safe, effective, high quality health service delivery. However, armed conflict and widespread violence can negatively affect the quality of health care through workforce shortages, supply chain disruptions and attacks on health facilities and staff. To improve IPC in these settings it is necessary to understand the specific barriers and facilitators experienced locally.

**Methods:**

In January and February of 2020, we conducted semi-structured interviews with hospital staff working for the International Committee of the Red Cross across eight conflict-affected countries (Central African Republic, South Sudan, Democratic Republic of the Congo, Mali, Nigeria, Lebanon, Yemen and Afghanistan). We explored barriers and facilitators to IPC, as well as the direct impact of conflict on the hospital and its’ IPC programme. Data was analysed thematically.

**Results:**

We found that inadequate hospital infrastructure, resource and workforce shortages, education of staff, inadequate in-service IPC training and supervision and large visitor numbers are barriers to IPC in hospitals in this study, similar to barriers seen in other resource-limited settings. High patient numbers, supply chain disruptions, high infection rates and attacks on healthcare infrastructures, all as a direct result of conflict, exacerbated existing challenges and imposed an additional burden on hospitals and their IPC programmes. We also found examples of local strategies for improving IPC in the face of limited resources, including departmental IPC champions and illustrated guidelines for in-service training.

**Conclusions:**

Hospitals included in this study demonstrated how they overcame certain challenges in the face of limited resources and funding. These strategies present opportunities for learning and knowledge exchange across contexts, particularly in the face of the current global coronavirus pandemic. The findings are increasingly relevant today as they provide evidence of the fragility of IPC programmes in these settings. More research is required on tailoring IPC programmes so that they can be feasible and sustainable in unstable settings.

## Background

Healthcare associated infections (HAIs) are the most frequent adverse outcome in healthcare delivery worldwide—at least one in 10 patients acquire an infection whilst receiving care in health facilities in low-and-middle-income-countries (LMICs) [1–4]. HAIs result in death, disability and costs to health systems and patients, whilst the increased use of antibiotics to manage them contributes to the spread of antimicrobial resistance [5]. Effective infection prevention and control (IPC) is crucial to ending avoidable HAIs and an integral part of safe, effective, high quality health service delivery [6]. The World Health Organization (WHO) estimates that effective IPC programmes can reduce HAI rates by 30% [7].

The WHO guidelines for IPC at the national and facility level, issued in 2016, outline eight core components for the implementation of effective IPC, to be applied across all countries and health facilities [8]. However, the feasibility of universal application varies greatly by context, and guideline adaptation must be informed by the barriers experienced in the local context.

In low-resource settings, challenges to the implementation of effective IPC programmes have been well documented. Hospitals often experience poor IPC governance at the national and facility level; a lack of political will translates into a scarcity of national level IPC policies, underfunding for IPC activities and dedicated staff, and resource shortages [9–14]. Additionally, many hospitals suffer from inadequate infrastructure, including poor water, sanitation and hygiene (WASH) facilities [9, 10, 14–16]. Challenges posed by staff shortages can be further hampered by a lack of IPC training for personnel and poor compliance with IPC practices, such as hand hygiene [9–16]. Overcrowding [9–12, 15] and inadequate infection surveillance systems [10, 11, 13, 14, 16] have also been documented as key constraints to effective IPC in low resource settings.

Conflict-affected settings represent another type of context in which IPC measures must be informed by the specific barriers and facilitators experienced locally. Whilst armed conflict and widespread violence generates an increased demand for emergency medical and surgical care, they also affect the determinants of health including food security, water and sanitation, and access to services [17]. The availability and quality of care in such settings can be further hampered by workforce shortages, supply chain disruptions and damage to health infrastructure [17–20]. The literature on issues relating to IPC in health care facilities in these settings is limited, but the available evidence suggests that HAIs are prevalent, and more specifically, that surgical site infections (SSIs) and antimicrobial resistance (AMR) are common complications [21–25]. Despite this, little work has been done to understand the challenges faced in these settings and what works to improve IPC at the facility level.

The qualitative research presented in this paper sought to explore the context-specific barriers to implementing successful IPC programmes in hospitals in conflict-affected settings, with a focus on how these settings differ from non-conflict, resource-limited settings, and what may work to improve IPC in these contexts. The research was undertaken in the period before the COVID-19 pandemic, and has implications for the current heightened need for effective IPC practices.

## Methods

Data were collected as part of a larger service evaluation to assess the status of IPC across 16 hospitals supported by the International Committee of the Red Cross (ICRC) in conflict-affected countries. This mixed-methods assessment compromised pre-questionnaire telephone calls, a hospital questionnaire, and semi-structured interviews. In this paper, we present findings from the semi-structured interviews. Information about the study sites draws upon the results from the questionnaire.

### Study sites

We selected twelve sites to participate in the interviews. Of the 16 hospitals included in the larger service evaluation, we excluded the three that had taken part in the pre-survey calls and a further one site for security reasons. Two of the twelve selected sites did not take part because of heavy workload during the study period. As such, we conducted ten interviews, and nine consented to inclusion in this analysis. Data collection took place across eight conflict-affected countries: Central African Republic, South Sudan, Democratic Republic of the Congo, Mali, Nigeria, Lebanon, Yemen and Afghanistan. All of the countries have experienced immense humanitarian suffering as a consequence of conflicts, such as mass displacement and refugee crises, widespread food insecurity, and major economic and health crises [26].

As one component of their humanitarian assistance within these countries, the ICRC provide emergency medical and surgical care to victims of armed conflict and other situations of violence [27]. All of the hospitals included within this study were either Ministry of Health (MoH) or private hospitals that were being provided with some level of support from the ICRC. In the majority of these facilities, the ICRC supports the provision of care on an existing structure which is determined by the local hospital management. Within this contract, a Memorandum of Understanding outlines the role and responsibilities of the ICRC team. Mobile ICRC teams, consisting of international staff, are present in all hospitals. At some sites, the ICRC directly employ local staff as well as working alongside local hospital employees. At the majority of the sites, the mobile ICRC teams are running surgical projects, and in some instances, they are also supporting other areas of the hospital such as obstetrics and gynaecology, paediatrics, general medicine, and the emergency department. At some sites, the ICRC are also responsible for training and capacity building. The level of financial support provided by the ICRC for the local hospital varied across the different sites. Some facilities were almost entirely supported by ICRC funds, including the provision of all resources (furniture, medical equipment and medicines) and the payment of local staff salaries/incentives. In other cases, the facilities received most of their funds from the MoH and ICRC funds were mainly used to support the specific ICRC activities.

Of the nine hospitals included, three were rural/field hospitals, three were secondary/county level hospitals, and three were tertiary/referral level hospitals. These hospitals differed in capacity, from 40 to 600 beds. They also differed in catchment population—some were the only hospital in a large area and as such treated a huge range of different medical and surgical patients, and others were more specialist sites, such as a field hospital that was specifically for combat-related injuries. A high proportion of the patients that the ICRC treated across all hospital levels were those requiring emergency care for weapon wounds.

The nine hospitals included in this study were very different with regard to their IPC programmes. Quantitative data from the wider service evaluation, specifically the hospital questionnaire, revealed that some hospitals lacked basic infrastructure and equipment for IPC, such as isolation rooms and materials for handwashing, and had staff with very low levels of education. Other hospitals had well established IPC programmes and supportive infrastructure for IPC, but fell down on areas such as infection surveillance. Areas that were particularly poor across a large number of facilities were the establishment of an IPC committee and the monitoring and audit of IPC practices.

### Study design and data collection

We conducted semi-structured interviews to explore the barriers to effective IPC through open-ended questions and probing [28]. Semi-structured interviews also enabled the data collection to build on issues that had been identified in the hospital questionnaire (that was part of the wider service evaluation). The original interview guides focused on three main areas: (1) the impact of conflict on the hospital, (2) challenges to IPC and (3) priorities for improvement. After one interview had been conducted, we added an additional section to the interview guides to be used with hospitals that had scored highly on the questionnaire, indicating they had elements of an IPC programme in place. In this, we asked how they achieved improvements to IPC and what advice they would give to other hospitals. The data collection was an iterative process and we adapted and improved interview guides throughout, for example by removing repetitive questions and rewording questions that were commonly misunderstood.

The nine semi-structured interviews presented in this paper were conducted remotely between January and February 2020 using a mobile instant messaging app. This method was selected by participants because it could be used with poor internet connection. Two members of the research team conducted eight of the interviews in English; one asked questions and one took notes. A third member of the research team conducted one interview in French (this interview was conducted with staff at a hospital in Mali). Before the interviews began, we provided participants with background information on the purpose of the interviews. All participants gave verbal consent to participate and be audio recorded. Interviews lasted between 45 and 75 min. We transcribed the interviews and those conducted in French were translated into English.

We sent an information sheet to all ten sites explaining the purpose of this analysis and describing how confidentiality would be upheld, which was of the utmost importance given the unique challenges of conducting research in conflict-affected settings. All references to specific hospitals and countries within quotes were removed, no participants were identified by name, and descriptions of the study sites were sufficiently abstracted to ensure they could not be identified by the services they provide or by their location. This study received ethical approval from the London School of Hygiene & Tropical Medicine (ref:22563).

### Respondents

All individuals who participated in the interviews were ICRC-funded staff, and selected as they were either the lead ICRC staff or their delegate; three hospital programme managers (HPM), two hospital administrators, two head nurses, one operating theatre nurse and one ward nurse/IPC lead. At three sites, the HPM was joined by a second staff member.

### Data analysis

We analysed the data thematically, drawing upon Braun and Clarke's Six Phases of Qualitative Analysis Framework [29]. Initial discussion of the transcripts between the research team focussed on recurring themes, similarities and differences between transcripts, and the likeness of the data to the existing literature. The transcripts were entered into NVivo 12 for coding, where deductive and inductive approaches were used in tandem [30]. An initial coding framework was developed from the literature on the challenges to IPC that exist in resource-limited settings and the first author applied these deductive codes to each transcript. The remaining coding followed an inductive approach, driven by the data itself, including conflict-specific data under the codes. The transcripts were coded until no new codes emerged and data under each code were cross checked by a second member of the research team. Following this, the codebook was refined, by removing and merging codes, before grouping codes into themes. Themes were discussed by three members of the research team. The final codebook contained 23 codes, grouped into 5 themes.

## Results

Participants described challenges to IPC that are explored along five key (and sometimes overlapping) themes: infrastructure and resources, management, the health workforce, visitors, and the direct effect of conflict on the hospital. Participants also spoke of recent IPC successes and strategies instituted to overcome challenges, which are presented within each of the themes. Hospitals are labelled A-I.

### Infrastructure and resources

Participants described that inadequate and poorly maintained buildings were a barrier to effective IPC. Damaged surfaces, including walls and floors, were difficult to keep clean. A head nurse at a hospital in sub-Saharan Africa described this problem: *'We have structural issues in the sense that the pavements are not flat and it’s difficult to clean them and keep them clean'* [hospital C]. They went on to describe that a lack of functioning windows and doors further hampered cleaning efforts: *'Even if we are cleaning every day there is always dust, insects, flies, mosquitoes… it’s difficult to manage'*.

As well as the physical structures in the building requiring maintenance, the layout and space within the hospitals also presented challenges to IPC. Inadequate bed capacity for the number of patients resulted in overcrowding on the wards; beds were too close together and patients had to occupy other available spaces such as on mattresses in the corridors and on the ward floors. As a consequence of this overcrowding, regular cleaning was problematic. A ward nurse from a hospital in the Middle East said that it was difficult to clean the rooms according to ICRC standards because *'the patients are like sardines laying there'* [hospital H]. Many of the hospital buildings also lacked sufficient isolation and cohorting rooms for infectious patients. Consequently, staff worried that outbreaks of HAIs would be more challenging to control.

Overcrowding on the wards was exacerbated when upsurges in conflict led to a sudden influx of patients. At hospital C, where the ICRC run a surgical ward for the treatment of those with weapon wounds, the head nurse described overcrowding as one of many ways that the conflict affected the services provided by the hospital: *'We have mass causalities and high numbers of patients and it becomes more and more difficult to manage hygiene and infection control with a huge number of patients'* [hospital C].

A number of sites lacked functional water points in patient care areas, meaning that critical IPC practices such as hand washing and environmental cleaning were more difficult to perform. Where there was no running water at handwashing stations, staff would have to collect water from the nearest source and carry it back to the ward, which was time consuming and not conducive to correct practices. A hospital administrator at a hospital in sub-Saharan Africa discussed the potential solutions for this in the context of cleaning: *'We need to ensure there is water available in the right places. If there's not, we could have trollies to help [the cleaners] carry the water'* [hospital B].

Waste management facilities were also lacking across the sites, with many reporting problems with the incinerators. Whilst some incinerators were broken, others were functional but there were no staff that had been trained on how to use them. At one particular hospital, an incinerator with inadequate capacity resulted in waste pilling up in the hospital grounds: *‘It's sharps, infectious waste, normal household waste, everything is one big pile. It's open, uncovered'* [hospital H].

A shortage of resources was another barrier to IPC across many hospitals and participants from across all hospital types acknowledged this. This included a scarcity of hospital furniture, as well as medicines and essential supplies for IPC such as PPE and hand hygiene and cleaning equipment. A hospital project manager running a surgical ward for weapon wounded in rural sub-Saharan Africa linked the lack of resources to increased infections: *'We always have the same problem – if they cannot access the right equipment, we see infections'* [hospital E]. Many participants explained that the ICRC would step in to provide supplies as a result of shortages, despite this not being part of the Memorandum of Understanding with the hospital management.

'The supportive role [the ICRC] play is in every element of the hospital. So it’s ‘supportive’ officially but we use this word loosely … ICRC are providing all the supplies and medical equipment, even chairs, tables, hand hygiene. We are providing everything as well as the incentives for the staff' [hospital B]

The conflict situation within some of the countries imposed an additional burden on the scarce resources. Strikes and security issues hindered the delivery and collection of essential supplies and resulted in supply chain breakdowns: *'We see the manifestations of instability – strikes, supplies arriving late because of strikes or security issues on the road'* [hospital C]. The conflict also resulted in supplies going missing. Some participants reported that things would be stolen from the hospitals and sold for money. At one hospital, the participant said that the money from stolen supplies could be used *'to send to the soldiers'*.

### Management

Management structures were frequently discussed by participants as having an impact on the success of an IPC programme. It was evident from the interviews that participants valued building a strong relationship with the hospital management. ICRC teams worked in a supportive role to the local hospital and as such, it was important to collaborate with hospital management to implement their respective programmes. This collective approach also worked towards ensuring the sustainability of the work that the ICRC were doing in the hospitals.

‘Our relationship with the hospital management is good. We have regular meetings and discuss together what the plan for the project is.’ [hospital F].

Sometimes, the conflict situation could make it difficult to foster such partnerships. One participant described that hospital management changed from one day to the next, meaning that it was difficult to institute IPC interventions across the hospital:

'The conflict affects everything in the hospital. One day things are red and the next day things are blue … the management is changing every day … [improving IPC] starts to become quite difficult.' [hospital H].

In addition to needing a strong relationship between hospital and ICRC management, participants said that an effective IPC governance structure was also critical to a successful IPC programme. Many participants placed great value on the establishment of an IPC committee or team that met regularly and represented all types of hospital staff. This enabled all the relevant personnel (hospital and ICRC staff) to discuss the IPC programme and set strategic targets based on which areas needed improving. Having an IPC governance structure in place meant that regular training could be implemented, which was also widely recognised as key to a successful IPC programme.

### The health workforce

A shortage of staff was a common across the sites. Some participants related this to a lack of funding, and others to the high numbers of patients and subsequent increased workload. A number of hospitals said that staff turnover was high due to the low wages, particularly amongst cleaning staff: *'There is turnover amongst [cleaners] … they come and go – if they find a motivation outside of the hospital that is better than what we offer them they quickly leave'* [hospital D]. Some hospitals were sent volunteers when staff numbers were low, but volunteers could be untrained. One participant described that the conflict in the country had a direct impact on the staff numbers: *'There are staff who say they cannot come to work because of war'* [hospital E].

Participants across all of the sites said that education levels amongst the health workforce were a challenge to IPC, in particular specific knowledge of IPC theory and practice. Some participants suggested that the conflict situation within the country had an impact on national training programmes.

In terms of capacity building, all participants believed that more in-service training was required for critical IPC practices. Participants indicated that cleaning staff in particular required tailored support because of low literacy levels, agreeing that on-the-job training was the most effective mode of delivery: “*We do a bit of theory but we mostly do practical work about how hygiene should be practiced. We simulate what they should do'* [hospital D]. A ward nurse at a rural hospital in the Middle East echoed this. They found that leading by example and demonstrating cleaning practices inspired staff to adopt these methods into their own daily practices.

However, participants acknowledged that training alone was insufficient to improve compliance to IPC practices. Hand hygiene and waste segregation were widely recognised as practices that needed continuous reinforcement. The head nurse at hospital in sub-Saharan Africa attributed this to a lack of motivation amongst staff: *'It’s not a lack of knowledge [about waste segregation] but it’s more related to motivation and a lack of adherence to practices'* [hospital F]. Participants commented that a lack of monitoring and follow-up after training resulted in limited changes in behaviour. New skills needed to be reinforced to be integrated into everyday practices. However, this could be challenging due to understaffing, as the head nurse of a surgical ward noted: *'We need to supervise but we are not always present'* [hospital C].

At sites where it was taking place, monitoring and supervision was used as a tool to assess progress towards IPC goals and increase motivation among the workforce:

'[The IPC team] monitor which services are better than others and this motivates services to work better. I think this is a good idea to push people to work harder. Monitoring of services is good as then you can know where they are and what needs to be done' [hospital E];

One hospital in particular spoke about the successes of the effective monitoring of services and supervision of staff. At a large hospital in sub-Saharan Africa, the nursing team noticed that health workers across different departments lacked a focal point for IPC information and support. In response to this, the IPC team elected a group of IPC champions (one nurse from each department). The IPC champions met regularly to discuss challenges and come up with solutions which they took back to their departments. They were also responsible for promoting and reinforcing correct practices and following-up new skills that had been taught in training. The head nurse noted:

'Since the implementation of the IPC champions there has been follow up with the cleaners of the hospital and supervision of their activities, working together in reinforcing cleaning … this is something that has started to improve … [Waste segregation] was something that before the implementation of the IPC champions wasn’t implemented in the hospital in all the different departments. Now we're seeing an improvement.' [hospital F].

### Visitors

Many participants identified large numbers of visitors as a challenge to IPC, despite some providing a critical role in caring for their sick relatives. Participants explained that visitors did not always understand the importance of hygiene practices, such as hand washing, which increased the risk of transmission of HAIs. At a hospital in sub-Saharan Africa, the hospital administrator acknowledged that it would be difficult to change such behaviours because it was not feasible to provide hygiene education to all of the patients and visitors that came through the doors.

In addition, large visitor numbers contributed to overcrowding on the wards, which hindered the cleaners' ability to effectively clean the wards.

'Visitors are making it harder to clean and harder to care for patients. We are dependent on caretakers to be there, the patients will not survive without them. The caretakers don't always understand the importance of hygiene' [hospital A].

On top of this, because of the volatility and tensions caused by the conflict, participants felt it was difficult to restrict visitors to the hospital and impose visiting hours: '*The overcrowding is a priority but it is also very hard to work on with the volatile situation here … It provokes aggression which could make the situation worse'* [hospital A]*.*

### Direct effect of conflict

Whilst some existing challenges to IPC were exacerbated by the conflict situation in the country, other challenges were a direct result of it. The general functioning of many hospitals was dependent upon the situation around them. Security issues were raised by a number of participants who explained that this often led to staff being evacuated, or even hospital closures.

A major challenge to IPC directly related to the conflict situation was the high rate of wound infection among patients. The majority of participants described how patients coming with weapon wounds often presented late and in many cases would already have infected wounds. One participant working on an ICRC ward for the weapon wounded in sub-Saharan Africa described the impact this had on IPC:

'The majority of our patients are coming late to the facility, I think the average is about 4–5 days [since injury] … They are coming already infected so the risk of transmitting infection to other patients is quite high' [hospital C].

Many participants also believed that these weapon wounds posed challenges to surgical site infection surveillance because it was difficult to assess where the original infection had come from, as a hospital administrator described:

'It’s difficult to assess post-operative infections … weapon wounds are often contaminated since the day they arrived. When there is a post-operative infection related to surgical care it is difficult to know the cause in an objective way' [hospital F].

## Discussion

This qualitative study found that inadequate hospital infrastructure, resource and workforce shortages, education of staff, inadequate in-service IPC training and supervision, and large visitor numbers are barriers to IPC in health facilities in conflict-affected settings, echoing research in other resource-limited contexts [9–16]. Our findings also highlight the unique challenges faced by hospitals in countries affected by conflict, in which high patient numbers, supply chain disruptions, high infection rates and attacks on healthcare infrastructures exacerbated existing challenges and imposed an additional burden on hospitals and their IPC programmes.

In our study, as within the wider literature [9, 10], many respondents saw the environment around them as unsupportive of IPC. The built environment and availability of resources play a critical role in supporting vital IPC practices, such as hand hygiene and environmental cleaning [31]. Inadequate buildings, WASH infrastructure and a scarcity of resources limited the opportunity for such practices in our study. The conflict situation in the country had an additional impact on the availability of supplies for IPC. Strikes and insecure roads resulted in supply chain disruptions; a well-documented challenge to health care provision in the context of armed conflict [17, 19].

Participants also felt that poor structural design led to overcrowding and an increased risk of HAI transmission. As a result of conflict and mass casualties, already crowded facilities could experience a sudden influx of patients, exceeding maximum capacity. Increased patients and visitors to the hospital made it harder for staff to follow IPC protocols, such as environmental cleaning, and general tensions and the volatile situation made imposing restrictions on visitors challenging. Participants felt that hygiene behaviours of patients and visitors (and sometimes staff), such as a lack of hand washing, also added to the risk of infection transmission. It is also worth noting that although in our study, and in other low- and middle-income settings [32], visitors and patient caretakers play a vital role in patient care, very little is known empirically about their impact on infection transmission. Overcrowding is acknowledged as a risk factor for increased HAIs [4] and an exploratory qualitative study in three tertiary hospitals in Bangladesh found that overcrowded wards and an uncontrolled flow of visitors were conducive to the transmission of infection [33]. Coupled with inadequate isolation capacity in many of the hospitals studied and high infection rates due to the nature of war wounds, participants in our study worried about the safety of the health care environment for patients, visitors and healthcare workers.

Low knowledge and education of staff and a lack of compliance to IPC practices stood out as a major challenge to IPC in our study. Similar barriers to IPC were found in a qualitative study among health workers in Mongolia, where staff had suboptimal knowledge of infection control, attributed to inadequate coverage of IPC in national training programmes [13].

Poor hand hygiene compliance was another barrier to IPC in our study; a challenge not unique to this setting and one known to play a central role in the transmission of HAIs globally [34]. Participants in our study felt that training alone was insufficient to improve compliance to critical IPC practices such as hand hygiene among healthcare workers. They emphasised the need for behaviour change interventions and monitoring and follow-up on top of in-service training. This echoes evidence from a review by Naikoba and Hayward [35], who found that stand-alone, educational interventions for hand hygiene had limited long-term impact on compliance. Instead, the most effective approaches were multifaceted, combining education with written material, reminders and continuous feedback. The implementation of WHO's multimodal improvement strategy [36] has been found to significantly improve hand hygiene compliance across a number of LMICs, including Mali [37] and Rwanda [38], demonstrating feasibility of such interventions in resource-limited settings.

Throughout the interviews, participants frequently referred to cleaning staff when discussing challenges to IPC. Respondents acknowledged their value to the IPC programme but stressed that they needed more training and supervision in order to improve their practice. Cleaners have often been neglected in IPC programmes—data from 56 health facilities across Zanzibar, The Gambia, Bangladesh and India found that less than half provided any form of IPC training for cleaners and that the cleaning workforce had low status and poor work conditions [39]. In our study, the need for locally adapted training materials that are tailored to cleaning staff with low literacy was evident, which is a priority recognised more broadly for IPC training in low-resource settings [12], and something that is being piloted through initiatives such as TEACH CLEAN [40].

In terms of what participants felt worked to improve IPC at their hospitals, value was given to the presence of an IPC committee, an essential first step in setting up an IPC programme [10]. Regular in-service training, including practical sessions and consistent monitoring and supervision of staff, which are both known to improve professional practice in health care settings [31, 41], were also recognised as strategies to improve IPC practices in our study. Of particular interest, with the potential to be transferred across ICRC sites and to other settings, was the implementation of IPC champions to support education and empower staff to make sustainable behavioural changes. The use of IPC champions as trainers and mentors is an approach recommended by WHO in their Core Components for Infection Prevention and Control at the healthcare facility [31]. Bundled IPC interventions have also seen success in low-resource settings, with potential to be implemented in conflict-affected settings such as those in this study [42, 43]. Across fifteen LMICs and 86 intensive care units, an intervention bundle including education, constant performance feedback and outcome and process surveillance improved IPC protocol adherence among staff and the incidence of the HAI under study [44], demonstrating the potential of low-cost and high-impact multi-component interventions in these settings.

Responses to the current coronavirus pandemic also provide important lessons for IPC programmes in resource limited and conflict-affected settings. One example includes the response to global PPE shortages, a challenge that was common for hospitals in our study before the onset of the pandemic. In such settings where supply chain disruptions and insecurity can result in inadequate PPE supply, the WHO’s guidance on the rational use, decontamination and reprocessing of PPE for covid-19 could be reviewed to create locally tailored solutions to mitigate the impact of PPE shortages [42].

Whilst participants in this study felt they had implemented approaches that were having a positive impact on IPC, many hospitals still faced the direct effects of conflict. Security incidents, hospital closures, changes in management and a lack of funding hampered progress towards IPC goals. The current global coronavirus pandemic has further highlighted the fragility of conflict and humanitarian settings, where global issues such as inadequate bed capacity and staff and PPE shortages during the pandemic, all identified as challenges to effective IPC in this paper, will be felt most acutely [46–48]. It emphasises the urgency of this topic and the need for additional research and intervention to strengthen the response for the most vulnerable populations.

An important strength of this study is that it is, to our knowledge, the first to explore qualitatively the barriers and challenges to IPC in hospitals in conflict-affected settings. While it highlights the stark challenges faced in such settings, similar to those identified by Mouallem and colleagues [48], it also offers realistic approaches to overcome some of these challenges in the context of limited resources and funding. It also presents an agenda for further research and intervention in these settings, particularly in the light of the global coronavirus pandemic. A limitation of this study is the relatively small sample, which limits the generalisability of our findings. A second limitation relates to the lack of data from local hospital staff. All of those who were interviewed were members of ICRC staff, some who had been working at the sites for a limited amount of time, and some who were not nationals to the country. Whilst highly experienced in their field, the data lacked the perspective of individuals who may have had greater knowledge and insight into the specific contexts in which they worked. Finally, relying on online methods for data collection through a messaging app posed a number of challenges. Poor connection resulted in disruption to the natural flow of conversation and the inability to use the video function, which at times made it difficult to build and maintain rapport with participants.

## Conclusion

Many of the barriers to effective IPC that were identified in this paper are common across LMIC settings. These include inadequate infrastructure, resource and workforce shortages, low workforce education levels, inadequate in-service IPC training, and large visitor numbers. In conflict-affected settings, there is an additional burden on health facilities and their IPC programmes. Upsurges of conflict and security incidents resulted in supply chain disruptions, high patient numbers, and high infection rates. While the hospitals included in this study faced significant barriers, they also demonstrated how they overcame certain challenges in the face of limited resources and funding. These strategies present opportunities for learning and knowledge exchange across contexts, particularly in the face of the current global coronavirus pandemic. While this study was carried out before the coronavirus pandemic was declared, the findings are increasingly relevant today as they provide evidence of the fragility of IPC programmes in these settings and how more research is required on tailoring IPC programmes so that they can be feasible and sustainable in unstable settings.

## Data Availability

The datasets generated and analysed during the current study are not publicly available due to the protection of individual privacy.

## Abbreviations

HAIs: Healthcare Associated Infections
IPC: Infection Prevention and Control
PPE: Personal Protective Equipment
WHO: World Health Organization
ICRC: International Committee of the Red Cross
LMIC: Low- and Middle-Income country
