# Correction to: Challenges and opportunities for infection prevention and control in hospitals in conflict-affected settings: a qualitative study

**DOI:** 10.1186/s13031-022-00433-5

**Published:** 2022-01-07

**Authors:** Hattie Lowe, Susannah Woodd, Isabelle L. Lange, Sanja Janjanin, Julie Barnet, Wendy Graham

**Affiliations:** 1 Department of Infectious Disease Epidemiology, Faculty of Epidemiology and Population Health, The London School of Hygiene and Tropical Medicine, London, UK; 2 Present Address: Institute for Global Health, Univeristy College London, London, UK; 3 Health Unit, International Committee of the Red Cross, Geneva, Switzerland

## Correction to: Conflict and Health (2021) 15: 94. 10.1186/s13031-021-00428-8

Following publication of the original article [1], the authors identified an error in the author name of Julie Barnet.

- The incorrect author name is: Julie Barnett
- The correct author name is: Julie Barnet

The author group has been updated above and the original article [1] has been corrected.
